# Post-ischemic inflammatory response in the brain: Targeting immune cell in ischemic stroke therapy

**DOI:** 10.3389/fnmol.2023.1076016

**Published:** 2023-04-03

**Authors:** Xueyang Shen, Mingming Li, Kangmei Shao, Yongnan Li, Zhaoming Ge

**Affiliations:** ^1^Department of Neurology, Lanzhou University Second Hospital, Lanzhou University, Lanzhou, China; ^2^Gansu Provincial Neurology Clinical Medical Research Center, The Second Hospital of Lanzhou University, Lanzhou, China; ^3^Expert Workstation of Academician Wang Longde, The Second Hospital of Lanzhou University, Lanzhou, China; ^4^Department of Cardiac Surgery, Lanzhou University Second Hospital, Lanzhou University, Lanzhou, China

**Keywords:** neuron, immune cell, glial, single-cell sequencing, ischemic stroke

## Abstract

An ischemic stroke occurs when the blood supply is obstructed to the vascular basin, causing the death of nerve cells and forming the ischemic core. Subsequently, the brain enters the stage of reconstruction and repair. The whole process includes cellular brain damage, inflammatory reaction, blood–brain barrier destruction, and nerve repair. During this process, the proportion and function of neurons, immune cells, glial cells, endothelial cells, and other cells change. Identifying potential differences in gene expression between cell types or heterogeneity between cells of the same type helps to understand the cellular changes that occur in the brain and the context of disease. The recent emergence of single-cell sequencing technology has promoted the exploration of single-cell diversity and the elucidation of the molecular mechanism of ischemic stroke, thus providing new ideas and directions for the diagnosis and clinical treatment of ischemic stroke.

## 1. Introduction

Stroke threatens human life and is one of the world’s most common causes of disability and death (Feigin et al., 2022). According to the Global Burden of Diseases 2019, the burden of stroke has increased significantly from 1990 to 2019 (70.0% increase in stroke events and 43.0% increase in stroke-related deaths) (Owolabi et al., 2022). Ischemic stroke accounts for 87% of all strokes globally (Virani et al., 2020). Acute ischemic stroke (AIS) is caused by a blockade of the local cerebral blood supply caused by various reasons, leading to hypoxia, ischemic changes in brain tissue, and a corresponding loss of nerve function. Since there is no effective treatment for neurological impairment caused by AIS except thrombolysis, early and accurate detection of ischemic injury is essential for initiating appropriate acute intervention and preventing recurrence strategies.

The current diagnosis of AIS is still based on clinical and neuroimaging evaluation (Bustamante et al., 2017). Therefore, it is essential to elucidate the pathophysiology of its pathogenesis further. The heterogeneity of cells in the central nervous system (CNS) has made it challenging to define the role of whole-brain cell subgroups in AIS pathogenesis and progression, which involves complex interactions between neurons, glial cells, and other cell types (Brioschi et al., 2021; Shen et al., 2021). Therefore, identifying changes in neuronal functions, transcription factors, and molecular pathways related to the above cellular processes might help to develop new therapeutic strategies. Therefore, it is necessary to understand each cell and its subgroup caused by AIS comprehensively. The traditional research method was to study the multicellular population and obtain the average value of a particular parameter. This method leads to a loss of heterogeneity information and many other “important details.” The population study can only obtain dominant cellular information for some complex tissues, such as highly heterogeneous solid tumor tissues. Some cells are ignored in the study because of their small size. Additionally, various microorganisms in nature are not only limited in number but also can not be expanded and cultured by traditional methods. Conventional techniques are of limited use for the analysis of these microorganisms, and therefore, single-cell RNA sequencing came into existence.

Single-cell sequencing mainly includes single-cell genome sequencing, single-cell epigenome sequencing, and single-cell transcriptome sequencing (scRNA-seq). There are currently many single-cell sequencing methods, such as Smart-seq, Drop-seq, and 10× genomics. Most have the same workflow: single cell isolation and collection, cell lysis, reverse transcription of RNA, cDNA amplification, library preparation, sequencing, and data analysis. The outstanding advantages of single-cell sequencing technology are that it can detect cell specificity and intercellular differences, explore the cooperative operation mode between cells, and study tissue heterogeneity from the perspective of a cell atlas. A combination of multi-omics analysis, cell and molecular imaging, and other technologies allows the generation of more accurate cell maps, deepens the understanding of disease development laws, helps find new targets for disease treatment and explores the cell development process. This article reviews the application of single-cell sequencing technology in AIS and its molecular basis in the pathological process of AIS.

## 2. Brain cellular damage

After an ischemic stroke, there are two zones of injury: the infarct core area and the ischemic penumbra (Heiss, 2000). Several brain cells in the ischemic penumbra, either dormant or semi-dormant, maintain the integrity of their morphology because of collateral arteries in the supply area after ischemia. Although these cells can continue to live in the brain for several hours, they cannot perform their original normal functions (Tang et al., 2022). Therefore, it is important to explicit the mechanism of ischemic cell death for the progression from penumbra towards irreversible injury. Guo et al. (2021) used scRNA-seq to comprehensively map the cell types of the ischemic penumbra in the middle cerebral artery occlusion (MCAO) mouse model and determined 24 cell clusters. All cells were divided into 13 major cell sets, and their marker genes have been confirmed in other articles. These cells include neurons (*MAP2*; Chen M.L. et al., 2021 and *TBR1*; Yook et al., 2019), astrocytes (*GFAP*; Wang et al., 2020 and *ALDOC*; Zeisel et al., 2015), microglia (*HEXB8*; Sousa et al., 2018), oligodendrocytes (*PLP1*; Marques et al., 2016), oligodendrocyte precursor cells (*PDGFRA*; Alessandrini et al., 2020), endothelial cells (*ITM2A*; Cegarra et al., 2022), pericytes (Desmin and *PDGFR-β*; Smyth et al., 2018), macrophages (*CD163*; Skytthe et al., 2020), B cells (*MS4A1* and *CD79A*; Lerman et al., 2022), T cells (*CD3*, *CD4*, *CD5*, *CD8*; Martinez-Lage et al., 2019), monocytes (*CD14* and *CD16*; Calderon et al., 2017), ependymal cells (*TTR*; Gokce et al., 2016), and fibroblasts (*COL1A1*; Bonney et al., 2022). Among them, most cells were microglia, astrocytes, and oligodendrocytes. The team of Lin and others obtained 3,186 mouse cerebrovascular cell samples from the GSE98816 dataset by examining the cerebrovascular-related databases (Kundishora et al., 2021). ScRNA-seq cell cluster analysis showed ten cell clusters and four cell subgroups, including endothelial cells, fibroblasts, oligodendrocytes, and microglia. After analyzing the differential expression of related genes in cell subgroups, it was the first time to find that calcium signaling pathway-related genes [*AC079305.10*, *BCL10*, BCL2 Related Protein A1 (*BCL2A1*), *BRE-AS1*, Dynein Light Chain LC8-Type 2 (*DYNLL2*), Epiregulin (*EREG*), and Prostaglandin-Endoperoxide Synthase 2 (*PTGS2*)] and transcription factors [Jun, Interferon Regulatory Factor 9 (IRF9), ETS Variant Transcription Factor 5 (ETV5), and Peroxisome Proliferator Activated Receptor Alpha (PPARa)] play a key role in AIS. Many cellular functions are regulated by calcium signals that are generated by different signaling pathways, and participate in the occurrence and development of many diseases (Madreiter-Sokolowski et al., 2020) such as Parkinson’s disease, Alzheimer’s disease, etc. *Jun* was identified to be associated with hypoxia in endothelial cells (Kundishora et al., 2021), *PPARA* is a transcription factor that regulates genes involved in fatty acid metabolism and activates hepatic autophagy, it is also an important factor regulating autophagy in the clearance of Aβ in Alzheimer’s disease (Luo et al., 2020). AIS leads to a significant increase in monocyte-derived cells (the proportion of cells changed from 2 to 16%), neutrophils, and pericytes, while endothelial cells and fibroblasts decreased slightly. The expression of specific ischemic injury-related genes, such as Glycerol-3-Phosphate Dehydrogenase 1 (*GPD1*) in oligodendrocytes, C-C Motif Chemokine Ligand 11 (*CCL11*) in pericytes, CD72 Molecule (*CD72*) and Leukocyte Immunoglobulin Like Receptor B4 (*LILRB4*) in microglia, were all upregulated (Zheng et al., 2021). Rusu et al. identify *GPD1* as a specific marker for dormant and chemoresistant brain tumor stem cells (BTSCs) and show that targeting *GPD1* disrupts BTSC maintenance and extends survival (Rusu et al., 2019). *CCL11* is important in the regulation of colitis and associated carcinogenesis (Polosukhina et al., 2021). These genes are expressed in ischemic stroke and other diseases. Biomarkers are signal indicators that are abnormal due to environmental pollutants at different biological levels (molecules, cells, individuals, etc.) before organisms are seriously damaged. It can provide early warning of serious toxic injury. The onset of ischemic stroke is acute, and the pathophysiological process is complex. Currently, the commonly used clinical biomarkers do not have the high specificity of the AIS, In the future, we can use single-cell sequencing technology to seek more specific biomarkers in ischemic stroke. Neurons in the cerebral cortex are mainly excitatory neurons and interneurons (Bandler et al., 2017). Excitatory neurons originate from the precursor cells of the developing cerebral cortex, while interneurons originate from the ganglion eminence (Shi Y. et al., 2021). By RNA sequencing, Zhong et al. (2018) analyzed more than 2,300 single cells in the developing human prefrontal cortex from 8 to 26 weeks of gestation, further dividing the excitatory neurons into seven subtypes. Among them, the 13 weeks of gestation is the critical period for the migration of the newly formed neurons. In the prefrontal cortex, the growth related gene expression of neurons increases at 16 weeks of gestation, and the functional gene expression, such as genes related to calcium input, increases at 19 weeks. At 19–26 weeks of gestation, genes related to axonogenesis were expressed in the prefrontal cortex, followed by genes related to synapse formation at 23–26 weeks, indicating that the initial formation of neural connections occurs between 19 and 26 weeks of gestation.

Interneurons were further divided into eight subtypes, and cell populations expressing interneuron markers, such as Transcription Termination Factor 1 (TTF1), LIM Homeobox 6 (Lhx6), and Distal-Less Homeobox 1 (DLX1), persisted throughout development. Calbindin 2 (Calb2) + and Somatostatin (SST) + interneurons appeared earlier, followed by Calbindin 1 (Calb1)+, Cholecystokinin (CCK)+, and Vasoactive Intestinal Peptide (VIP) + interneurons. Overall, the developmental peak of excitatory neurons appeared at 16 weeks of gestation, and that of interneurons appeared at 26 weeks. There are potential subtype-specific marker genes in each neuronal cluster (Zhong et al., 2018). Chen et al. (2017) used scRNA-seq to demonstrate the diversity of hypothalamic cells. They identified 15 glutamatergic neuron subtypes (Glu1-Glu15), 18 γ-aminobutyric acid (GABA)-ergic neuron subtypes (GABA1-GABA18), and one histaminergic neuron cluster (Hasta) expressing high levels of Histidine Decarboxylase (HDC). Kiss1 and Pomc represent Glu11 and Glu13 cell clusters, respectively, while Vip and Agouti Related Neuropeptide (Agrp) represent GABA9 and GABA15 cell clusters, consistent with their roles in controlling neuronal differentiation and identity. Recent studies have shown that RNA binding proteins CUGBP Elav-Like Family Member 1/2 (CELF1/2), Muscleblind Like Splicing Regulator 2 (Mbnl2), and KH RNA Binding Domain Containing, Signal Transduction Associated 3 (Khdrbs3) are preferentially expressed and more active in glutamatergic neurons (Feng et al., 2021). In contrast, ELAV Like RNA Binding Protein 2 (ELAVL2) and QK are preferentially expressed and more active in GABAergic neurons, indicating the hierarchical regulation of alternative splicing between different neuronal cell types and providing a basis to specify the identity and function of neurons. Single-cell sequencing studies revealed the heterogeneity among different neuronal and non-neuronal cells in various brain regions (Zeisel et al., 2015). Neuronal changes in some neurological diseases have not been described in detail. Moreover, the changes and migration of neuronal cells in the ischemic penumbra have not been reported, and therefore, further research is needed for a better understanding (Table 1).

**Table 1:** Application of single cell sequencing technology in the study of ischemic stroke.

Study name and years of publication	Methodology	Sample source	Number of cells sequence	Molecules/pathways identified
Chen et al. (2017)	Seurat	Mice	14,000	Defines 11 non-neuronal and 34 neuronal cell clusters with distinct transcriptional signatures on hypothalamus
Zhong et al. (2018)	Seurat and forest	Human embryonic PFCs	2,309	Found the intrinsic development-dependent signals that regulate neuron generation and circuit formation
Wang et al. (2020)	HiSeq 3000	Mice	unknown	Astrocyte-derived E2 is also critical for the activation of LIF/JAK/STAT3 signaling in astrocytes, which is a key regulatory pathway responsible for astrocyte activation
Guo et al. (2021)	10× Genomics	Mice	18,273	Identified 13 cell types and subtypes in the ischemic stroke penumbra
Lin et al. (2021)	Seurat (v. 4.0.4) and singleR (v. 1.0)	Mice	19,922	Calcium signaling pathway-related genes (AC079305.10, BCL10, BCL2A1, BRE-AS1, DYNLL2, EREG, and PTGS2) and TFs (JUN, IRF9, ETV5, and PPARA) were identified to play a key role in IS
Zheng et al. (2021)	10× Genomics	Mice	58,528	Identified 17 cell clusters
Feng et al. (2021)	Quantas pipeline	Mice	22,578	Distinct splicing programs between glutamatergic and GABAergic neurons and between subclasses within each neuronal class
Xie et al. (2021)	10× Genomics	Human glioblastoma and peritumoraltissue	97,584	ECs in GBM are associated with partially intact BBB phenotype characterized by downregulation of transporter genes and upregulation of transcytosis gene
Shi L. et al. (2021)	Seurat 2.3.4 and DESeq2	Mice	10,925	Treg cell-derived osteopontin acted through integrin receptors on microglia to enhance microglial reparative activity, consequently promoting oligodendrogenesis and white matter repair
Shi X. et al. (2021)	10× Genomics	Mice	12,000	Deleting MEGF10 and MERTK phagocytic receptors, inhibiting phagocytosis of microglia/macrophages or astrocytes in ischemic stroke improved neurobehavioral outcomes and attenuated brain damage
Cho et al. (2022)	NovaSeq 6000	Human peripheral blood	26,302	Demonstrating the increased number of NK cells and new monocyte subclusters of mild ischemic stroke
Ma et al. (2022)	10× Genomics	Mice	104,382	The astrocytes tend to play an independent role in neuroprotection or adaption in the local ischemic-reperfusion regions at 12 h
Li et al. (2022)	10× Genomics	Mice	18,755	The first scRNA-seq data set for immune cells in the stroke aged brain

## 3. Participate in inflammatory reaction

Under normal circumstances, the human CNS is separated from the peripheral immune system through a complete blood–brain barrier. The nerve cells die of ischemia within a few minutes after an ischemic stroke, releasing “danger signals” and activating the innate immune response in the brain. Promote the production of neurotoxic substances, such as inflammatory cytokines, chemokines, reactive oxygen species and nitric oxide (Shi et al., 2019), and the destruction of the blood–brain barrier is mediated, resulting in a series of inflammatory cascades. At the same time, the expression of adhesion molecules in the cerebral vascular endothelial cells increases, and immune-inflammatory cells such as polymorphonuclear neutrophils, lymphocytes, and monocyte macrophages enter the brain tissue through vascular endothelial cells. By recognizing the antigens exposed by the CNS in the brain, the immune-inflammatory cells activate the adaptive immune response, further mediating the secondary injury of neurons and aggravating the neurological defect. At the same time, to reduce the damage mentioned earlier, the body initiates peripheral immunosuppressive response after the stroke through negative feedback, thereby increasing the incidence of post-stroke infection (Iadecola et al., 2020). Different immune cells play different roles after the occurrence of AIS. Understanding the type, migration, and transformation of cells would help explore the pathogenesis of AIS further and find new intervention targets.

### 3.1. Microglia

Microglia play a key role in brain development, immune defense, and maintenance of CNS homeostasis (Lehnardt, 2010). Common immunological markers of microglia include CD45, CD68, HLA-DR, and IBA-1, which are slowly updated at an average rate of 28% per year. Only 2% of microglia are believed to proliferate at a specific time (Askew et al., 2017; Réu et al., 2017). Previous studies exhibited a wide range of microglial DEGs between large tissues and single cells (>50%), indicating that microglia are a core player in ischemic stroke inflammation. Meanwhile, microglia have 157 unique DEGs in all cell types, ranking first, followed by monocyte-derived cells, oligodendrocytes, endothelial cells, and CNS-related macrophages (Zheng et al., 2021). Guo et al. also confirmed that microglia accounted for the most significant number of cells after MCAO induction. Using scRNA-seq, they also showed that microglia exhibit polarization and differentiation in two different progression trajectories 24 h after MCAO (Guo et al., 2021). The most enriched signatures in subclusters 3, 4, 9, and 10 were the hypoxia pathway, as well as TNF-α, IL-6, and IL-2 genes and pathways related to inflammation. Compared with the previous M1/M2 dichotomy of microglia polarization, it would be of great significance to further study the multi-polarization of microglia by using single-cell technology. On the other hand, Li et al. found six subpopulations of microglia in aged rats after ischemia–reperfusion (Li et al., 2022). MG5 and MG6 were the main subpopulations after stroke. In MG5 cells, the expression of Top2a, Stmn1, Mki67, and Cdk1 was significantly upregulated, indicating that MG5 cells were in a highly proliferative state. Compared with MG1, expression of the steady-state genes of microglia (such as P2ry12, Tmem119, Cx3cr1, and Hexb) were downregulated in MG5. While MG6 represented a unique microglial state that appears only after stroke, was close to neutrophils on the uniform manifold approximation and projection (UMAP) map, and expressed high levels of Cxcr2, S100a8, Il1b, and Mmp9, showing a unique “neutrophil-like” phenotype and participating in the inflammatory response. Zheng et al. detected CCL7 and CCL12 in microglia, providing evidence for the molecular and cellular basis of inflammatory response after MCAO induction. They showed that the release of damage-associated molecular patterns (DAMPs) in damaged tissues initiated the secretion of these chemokines and cytokines by glial cells (Li P. et al., 2018; Zheng et al., 2021). CCL22 and many other inflammatory factors further exacerbated brain injury by enhancing cytotoxicity. Chemotactic factors direct the migration of immune cells, multipotent stem cells, and progenitor cells under physiologic and pathologic conditions. CCL7 is also highly expressed in the tumor microenvironment of various cancers, including colorectal cancer, breast cancer, oral cancer, renal cancer, and gastric cancer (Lee and Cho, 2020). CCL12 and CCL20 also contributes to the progression of many cancers, such as liver cancer, breast cancer (Chen et al., 2020; Li B.H. et al., 2020), etc. Similarly, some studies showed that in the MCAO group, the intercellular interactions dominated by microglia and macrophages increased significantly after AIS, especially the interactions between microglia and other immune cells, astrocytes, pericytes, and oligodendrocytes (Zheng et al., 2021). These findings imply that 24 h before the onset of AIS might be a good window for intervention to help cells survive.

### 3.2. Macrophages

Compared with microglia, the polarization of peripheral macrophages seems complete, indicating that microglia and blood immune cells in the brain have different activation periods after AIS (Zubova et al., 2022). It has been recently found that the infiltration and polarization of macrophages can be detected in the early stage of AIS, thus promoting the progress of inflammation (Bernstein and Rom, 2020). Zheng et al. identified six macrophage subpopulations in the MCAO group, all expressing core characteristic genes *LYV1*, *CD163*, *MRC1*, and *CBR2* (Zheng et al., 2021). According to the changes in the cell proportion of the six subpopulations after ischemia injury, it was found that the fourth and fifth subpopulations of macrophages were mainly from the MCAO group. The MHCII-related antigen-presenting molecules of the fourth subpopulation (such as H2-Aa, H2-Ab1, and Cd74) were higher. The fifth subpopulation was characterized by increased expression of genes related to oxidative phosphorylation and respiratory electron transport chain, such as Cox7b, Cox8a, and Uqcr11. Therefore, obstructing the initial recruitment of peripheral immune cells might be an effective measure to alleviate stroke inflammation.

### 3.3. Neutrophils

Neutrophils are short-lived but influential immune cells that can provide an early and robust inflammatory response after tissue injury, including AIS (Aronowski and Roy-O'reilly, 2019). Neutrophils are also one of the most abundant cell populations in the injured brain, and their number rapidly peaks at the lesion site 1 to 3 days after the occurrence of AIS (Grønberg et al., 2013). At the same time, Neutrophils could also worsen AIS through multiple mechanisms, including physical blockade within microvessels to reduce cerebral blood flow further and direct entry into the brain parenchyma, followed by the release of particles containing antimicrobial enzymes and chemical components, such as MMP9 that may further damage brain tissue. scRNA-seq provides a unique insight into the cellular heterogeneity of inflammatory response after AIS, revealing immune cell subpopulations with different functions in the pathophysiology of ischemic stroke and seeking a better target for inflammatory intervention in the subsequent stroke.

### 3.4. Monocytes

Cho et al. used individual peripheral blood mononuclear cells (PBMCs) prepared using ddSEQ (Illumina BioRad) and sequenced on the Illumina NovaSeq 6000 platform (Shi X. et al., 2021). They found that the overall gene expression of NK cells in AIS patients showed a strong increase in cell activity and a significant decrease in the number of CD14+ monocytes subdivided into dendritic cells and CD14+ monocytes associated with NK cells. Patients with mild to moderate AIS show a slight increase in the proportion of NK cells in the blood on day 7 (Yan et al., 2009), but patients with moderate to severe stroke do not exhibit any changes in the proportion of NK cells (Peterfalvi et al., 2009; Jiang et al., 2017). Reduced NK cell numbers are associated with reduced cytokine levels in the blood. Cytokine deficiency in the blood of patients with ischemic stroke leads to immunosuppression and post-stroke infection (Wang et al., 2019). This is a severe complication leading to poor outcomes of AIS, and previous reports have also shown that NK cells are involved in this process (Chen et al., 2019; Wang et al., 2019). Gene set variation analysis (GSVA) showed that oligodendrocytes contained nine subpopulations rich in IL-6, complement system, TNF-α Pathway, and KRAS signaling, indicating that serum-and glucocorticoid-inducible kinases 3 (SGK3) from oligodendrocytes may play an essential role in regulating oligodendrocyte viability and inflammatory response in the acute phase of ischemic stroke (Inoue et al., 2016).

### 3.5. Others

**Figure 1:**
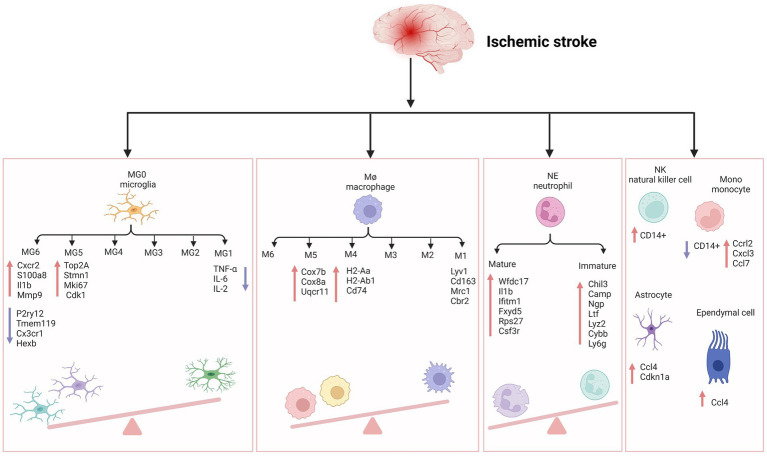
The inflammatory reaction of ischemic stroke. MG, microglia; Cxcr2, cxc chemokine receptor 2; Il1b, interleukin-1 beta; Mmp-9, matrix metallopeptidase 9; P2ry12, recombinant purinergic receptor P2Y, G protein poupled 12; Tmem119, transmembrane protein 119; Cx3cr1, c-x3-c motif chemokine receptor 1; Hexb, hexosaminidase subunit beta; Top2A, DNA topoisomerase II alpha; Stmn1, stathmin1; Mki67, marker of proliferation ki-67; TNF-α, tumor necrosis factor-α; IL-6, interleukin- 6; IL-2, interleukin-2; Cdk1, cyclin-dependent kinases 1; Cox7b, cytochrome c oxidase subunit 7b; Cox8a, cytochrome c oxidase subunit 8a; Uqcr11, ubiquinol-cytochrome c reductase, complex III subunit XI; H2-Aa, histocompatibility 2, class II antigen A, alpha; H2-Ab1, histocompatibility 2, class II antigen A, beta 1; Mrc1, mannose receptor C-Type 1; Cbr2, carbonyl reductase 2; NE, neutrophil; Wfdc17, WAP four-disulfide core domain 17; Ifitm1, interferon induced transmembrane protein 1; Fxyd5, FXYD Domain Containing Ion Transport Regulator 5; Rps27, Ribosomal Protein S27; Csf3r, colony stimulating factor 3 receptor; Chil3, Chitinase-3-like protein 3; Ltf, lactotransferrin; Lyz2, lysozyme 2; Cybb, cytochrome b-245 beta chain; Ly6g, lymphocyte antigen 6 complex locus G6D; NK, natural kill cell; Mono, monocyte; Ccrl2, c-c motif chemokine receptor like 2; Cxcl3, c-x-c motif chemokine ligand 3; Ccl7, c-c motif chemokine ligand 7; Ccl4, c-c motif chemokine ligand 4; Cdkn1a, cyclin dependent kinase inhibitor 1a. Figure was created with Biorender.com.

Zheng et al. detected an upregulation of CCL7 and CCL12 in microglia, CCL4 and CDKN1a in astrocytes, and CCL4 in ependymal cells, providing evidence for the molecular and cellular basis of inflammatory response after MCAO. They showed that the release of DAMPs in damaged tissues initiates the secretion of these chemokines and cytokines by glial cells (Li P. et al., 2018; Zheng et al., 2021). CCL7, CCL12 and CCL4 are proinflammatory chemokines belonging to the CC family, and their expression is not specific in ischemic stroke. Increase in mRNA and protein levels of CCL4 in the animal model of temporal lobe epilepsy (Guzik-Kornacka et al., 2011). Subsequently, many infiltrating monocytes and lymphocytes were characterized by increased expression of CCRL2, CXCL3, CCL7, and CCL22, and many other inflammatory factors further exacerbated brain injury by enhancing cell excitotoxicity. Furthermore, AIS reduces the correlation between fibroblasts and other cells (Zheng et al., 2021). Similarly, 24 h after ischemia–reperfusion, astrocytes can act as a signal amplifier to release inflammatory signals such as cytokines and attract assistance from distal sites (Figure 1; Ma et al., 2022).

We have checked the Figure 1 carefully and corrected the “I11b” into “Il1b” in the MG6 part.

## 4. Destroy the blood–brain barrier

The blood–brain barrier (BBB) is a highly selective semi-permeable border of brain microvascular endothelial cells regulated by tight junctions. Pericytes, basement membranes, and glial cells induce and maintain the essential functions of the blood–brain barrier. The complex they form governs the movement of molecules, ions, and cells between the blood and CNS through interaction so that the blood–brain barrier can strictly regulate CNS homeostasis (Daneman and Prat, 2015; Profaci et al., 2020). This complex also plays an essential role in maintaining the physiological function of neurons and protecting the CNS from toxins, pathogens, inflammation, and damage. After the occurrence of AIS, activated immune cells successively reach the ischemic area through the breakdown of the blood–brain barrier. They could play a double-edged sword role by destroying or protecting the integrity of the blood–brain barrier (Li Y. et al., 2018).

### 4.1. Endothelial cells

Endothelial cells (ECs) are critical cellular components of BBB. Brain ECs establish a continuous complex of tight and adhesive junctions along the EC-EC contact, providing a size-selective barrier that can further express different inflow and outflow transporters. Brain ECs also have a deficient level of vesicle transport, further limiting the passage of blood-derived water-soluble molecules of various sizes in the blood (Daneman and Prat, 2015). Zheng et al. Identified six endothelial cell subpopulations in the MCAO group using scRNA-seq. Ischemia-induced inflammation and oxidative stress increased the death of endothelial cells, thus decreasing the cell proportion of the MCAO group. In contrast, the cells of BBB-related clusters increase, including a series of BBB functional disorder module-related gene expressions, such as ADAMTS4, UPP1, TIMP1, and PDLIM1 (Munji et al., 2019).

Moreover, BBB enriched endothelial cell subpopulation 3 highly expresses the *KRAS* gene and participates in the regulation of the RAS/MAPK pathway, which is closely related to the apoptosis of cerebral microvascular endothelial cells in AIS (Yong et al., 2019). ScRNA-seq of human glioblastoma showed that ECs in the surrounding tissues have a quiescent phenotype characterized by high expression of BBB-enriched genes, including SLC2A1 and KLF2 (Xie et al., 2021). KLF2 is a crucial transcription factor that coordinates a gene network that promotes EC response to blood flow (Dekker et al., 2006) and is one of the top ten enriched genes in the brain EC cluster. GLUT1 encoded by *SLC2A1* is highly expressed in BBB ECs and promotes glucose transport across the BBB (Yong et al., 2019). Depleting GLUT1 in adult brain ECs leads to inflammation and activation of extracellular matrix-related genes (Veys et al., 2020). These results contradict the common belief that glioblastoma EC has a partially intact BBB phenotype characterized by the downregulation of transporter genes. At the same time, the expression of linker molecules remains normal or increased, and the vascular marker of BBB destruction—plasmalemma vesicle-associated protein (PLVAP) is in a high expression state.

### 4.2. Glial cells

The glial cells in the CNS mainly include astrocytes, microglia, and oligodendrocytes. Different glial cells are interconnected to neurons and surrounding blood vessels, forming a complex information exchange network (Huang et al., 2019). Glial cells support nerve transmission, maintain extracellular ion balance, insulate axons, and accelerate electrical signal transmission (Allen and Lyons, 2018). Guo et al. showed that in the MCAO group, Cyr61 in astrocytes and Sgk3 in oligodendrocytes were overexpressed (Guo et al., 2021). These genes might be potential therapeutic targets in this stage of AIS. Overexpression of the *CYR61* gene may contribute to the survival of astrocytes after AIS. Although there is a lack of research on AIS, previous studies have shown that the *CYR61* gene is closely related to stress and tumor cell proliferation (Sun et al., 2020). A study examining the relationship between astrocytes and BBB in heat stroke rats showed that astrocytes, but not neuronal DEGs, were rich in clusters of leukocyte chemotaxis and cytokine signals as cytokines/chemokines, toll-like receptors, and NF-κB signaling pathway (Niu et al., 2017). These chemokine-related genes were not found in neurons but combined into a regulatory sub-network in the protein–protein interaction network of astrocyte DEGs. In primary cultured astrocytes, scRNA-seq and qPCR showed upregulation of C6, CCL3, and CCR1 after heat stress but downregulation in heat stroke rats (Niu et al., 2017), while the transcriptional levels of CCL3 and CCR1 were downregulated in heat stroke rats (Audet et al., 2016). Glial cells are one of the essential components of BBB. Further exploring the regulatory mechanism of BBB injury might be one of the targets of AIS intervention in the future.

### 4.3. Others

**Figure 2:**
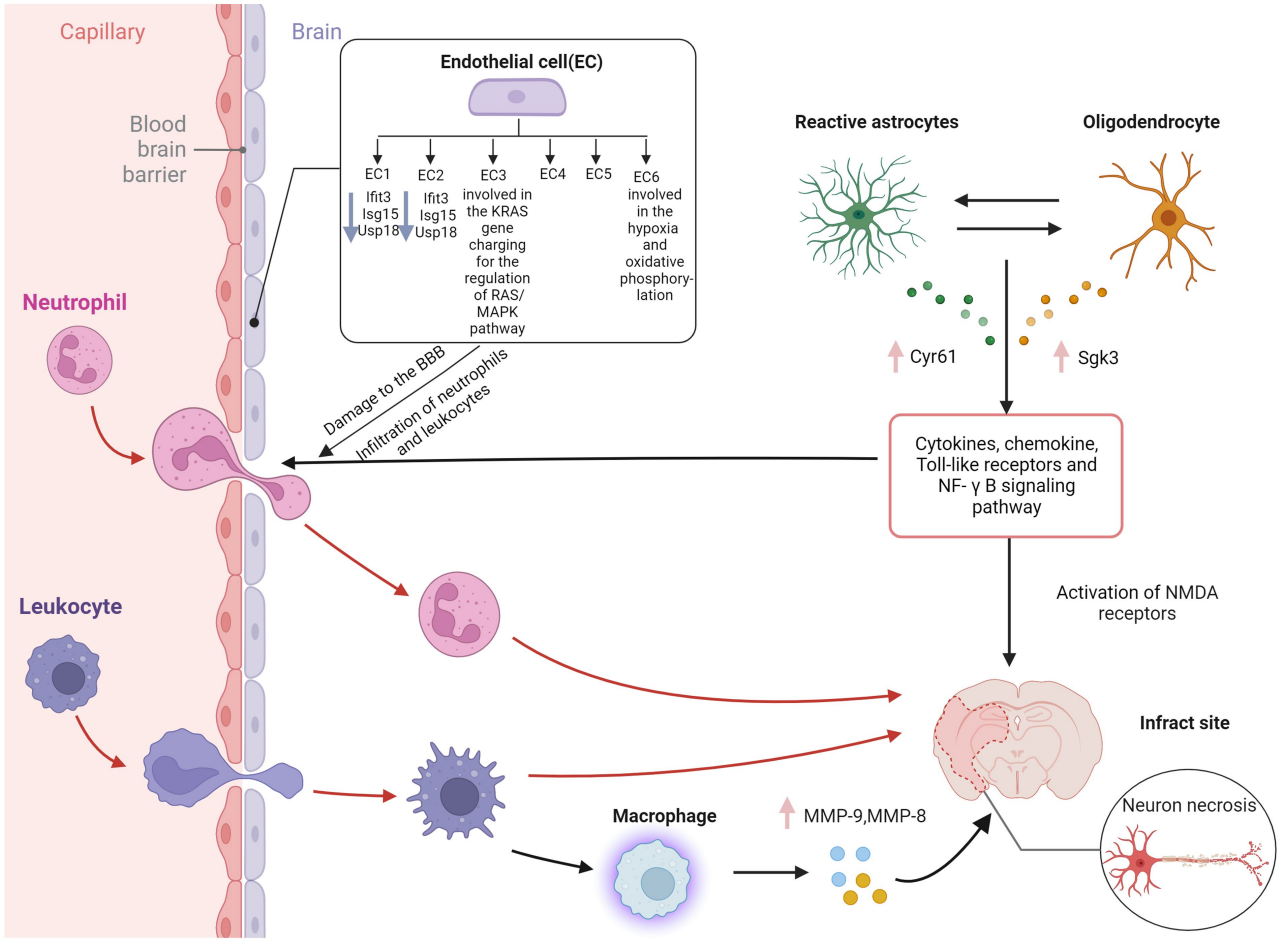
Blood–brain barrier disruption in ischemic stroke. EC, endothelial cell; IFIT3, interferon induced protein with tetratricopeptide repeats 3; Isg15, interferon-stimulated gene 15; USP18, ubiquitin-specific protease 18; Cyr61, cysteine-rich protein 61; Sgk3, serum and glucocorticoid induced kinase; MMP-9, matrix metallopeptidase 9; MMP-8, matrix metallopeptidase 8; NF-κB, nuclear factor kappa-B; NMDA receptor, N-methyl-D-aspartic acid receptor; BBB, blood–brain barrier. Figure was created with Biorender.com.

Other immune cells also play an essential role in BBB injury. Neutrophils cause the breakdown of the blood–brain barrier by releasing MMP9 and other substances, further aggravating neuroinflammation (Amantea et al., 2009). MMP9 has the ability to degrade the extracellular matrix components and has important role in the pathophysiological functions (Mondal et al., 2020). Some studies have shown that blood neutrophils in AIS patients increase and are closely related to the severity of AIS, infarct volume, and worse neurological function (Kim et al., 2012). In addition, peripheral macrophages are generally recruited to the lesion through the damaged BBB within 24 h (Jian et al., 2019). Previous studies have shown that MMP9 is highly expressed in microglia, leading to hydrolysis and vascular damage, thereby causing changes in BBB (Rosenkranz et al., 2020). Another study showed that MMPs could induce the physical destruction of BBB by digesting BBB matrix proteins (Ji et al., 2017). Guo et al. showed that MMP9 and MMP8 were overexpressed in macrophages of the MCAO group (Guo et al., 2021), indicating that in addition to microglia in the ischemic penumbra, the breakdown of BBB may also be caused by macrophages recruited from the periphery. Compared with microglia and macrophages, subpopulations of astrocytes and oligodendrocytes showed less polarization in the early stage of AIS in scRNA-seq analysis. Inflammatory mediators such as cytokines and chemokines are released through the extracellular BBB when astrocyte gap junctions are damaged during AIS (Ma et al., 2018). The mechanism of cerebral edema after cerebral ischemia is complex, caused by the interaction between many factors, and can lead to high mortality. Therefore, effective treatment measures are necessary to improve the functional prognosis of patients with cerebral ischemia (Figure 2).

## 5. Promote nerve repair

In the recovery stage of ischemic stroke, microglia, astrocytes, and NG2 glia proliferate highly, forming reactive gliosis and glial scars in the lesion area (Wanner et al., 2013). Glial scars have traditionally been thought to hinder axon regeneration and myelin sheath regeneration (Fawcett and Asher, 1999). However, there is increasing evidence that the formation of glial scars also contributes to CNS axons regeneration (Rolls et al., 2009; Anderson et al., 2016).

### 5.1. Microglia

Studies show that microglia and astrocytes are activated in mice with ischemic brain injury to form glial scars (Shi X. et al., 2021). The phagocytic capacity of these glial cells was enhanced in the scar area, and more synapses were phagocytized. The phagocytic ability of microglia was more substantial than that of astrocytes. High-resolution transmission electron microscopy further confirmed that synapses existed in the cell bodies of microglia and astrocytes. The GSVA results of the sixth and eighth subpopulations of microglia by Guo et al. showed that these subpopulations are MG2 type, mainly rich in the KRAS signaling pathway (Heiss, 2000), and are closely related to the survival of cancer cells (Saad et al., 2019), and may also be related to repair, neurogenesis, axon remodeling and angiogenesis (Hu et al., 2015).

### 5.2. Astrocytes

ScRNA-seq analysis revealed ten different astrocyte subpopulations with different cellular functional characteristics, and synaptic pruning-related processes significantly upregulated the astrocyte transcriptomic characteristics of subpopulation three. During the post-AIS repair and remodeling phase, reactive microglial proliferation and astrogliosis actively phagocytize synapses through MEGF10 and MERTK-related pathways and inhibit microglial proliferation or astrogliosis-mediated synaptic phagocytosis by improving the prognosis of AIS mice (Shi X. et al., 2021). MEGF10-knockout mice show defective long-term synaptic plasticity and impaired formation of hippocampal memories (Lee et al., 2021). Previous studies have shown that hypoxia and focal cerebral ischemia increase the number of neural stem/progenitor cells (NS/PCS) in the hippocampal and subependymal area of MCAO mice after 1 month with an increase in neuroblasts. The canonical Wnt signaling pathway is involved in this process (Knotek et al., 2020). Kraska et al. evaluated the effect of the classical Wnt signaling pathway on the differentiation potential of NS/PCS under physiological conditions and after ischemia (Kriska et al., 2021). They showed that focal cerebral ischemia increased the expression of target genes and cell type-specific proteins in the Wnt signaling pathway, affected the electrophysiological characteristics of differentiated NS/PCS, and promoted neurogenesis. ScRNA-seq provided an essential clue for analyzing the role of the Wnt signaling pathway in patients with ischemic stroke. A recent study found that astrocytes play an independent neuroprotective role 12 h after ischemia–reperfusion, mainly manifested by the activation of oxidative phosphorylation, gap junction and tight junction, ferroptosis, and other pathways (Ma et al., 2022).

### 5.3. Treg cells

**Figure 3:**
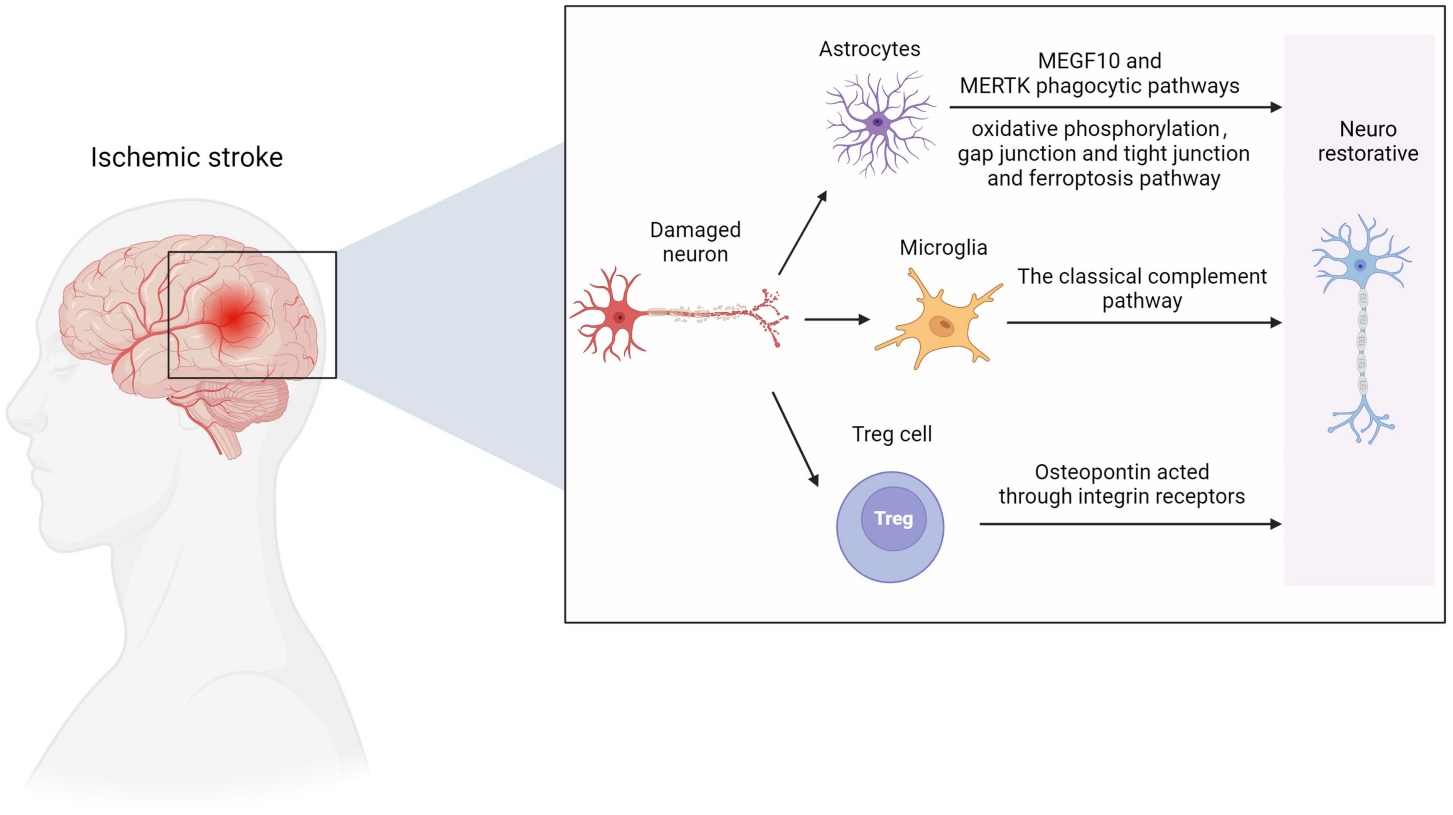
Nerve repair in ischemic stroke. MEGF10, multiple EGF like domains 10; MERTK, MER proto-oncogene, tyrosine kinase. Figure was created with Biorender.com.

Using scRNA-seq and flow cytometry, Shi et al. showed that the number of Treg cells in the brain significantly increased from 1 to 5 weeks after MCAO mouse modeling (Shi L. et al., 2021). Treg cells-derived osteopontin acts through integrin receptors on microglia to enhance the repair activity of microglia, thus promoting oligodendrocytes regeneration and white matter repair in the chronic phase of AIS. A recent study further confirmed that Treg cells contribute to the recovery of the CNS in the chronic phase (>1 week) after the initial ischemic injury. The number of Treg cells in the ischemic brain increases for at least 1 month after stroke. These accumulated Treg cells are thought to promote functional recovery after stroke by inhibiting astrocyte proliferation (Ito et al., 2019). Additionally, Treg cell depletion inhibits the proliferation of neural stem cells 4 days after stroke, indicating the involvement of Treg cells in neurogenesis (Wang et al., 2015). These findings reveal that Treg cells are neural repair targets for AIS recovery. In the early stage of ischemia, endothelial progenitor cells can replace the damaged vascular endothelial cells and remodel the blood–brain barrier. The release of various nutritional factors can also protect other damaged cells. In the recovery period of cerebral infarction, vascular regeneration, neuroprotection, and nerve regeneration complement each other, and its potential mechanisms include neovascularization, which provides blood flow with nutrients. Endothelial progenitor cells can secrete chemical factors such as SDF-1 and VEGF to create a microenvironment suitable for nerve regeneration and survival (Chen B. et al., 2021). In addition, endogenous neural stem cells migrate to the periphery of the infarct along the newly formed blood vessels, promoting nerve regeneration (Figure 3).

## 6. Limitations

There are still many limitations in single-cell sequencing, which cause data bias and distortion: (1) Batch effect during technical operation (Li X. et al., 2020); (2) Cells get lost during preparation of single cell suspension (Xin et al., 2016); (3) Low cell activity leads to the failure of the reverse transcription (Li L.C. et al., 2018); (4) RNA with low abundance gets lost during reverse transcription (Wang et al., 2016); (5) Changes in gene transcriptome during the preparation of single cell suspension (Wang et al., 2016). Since the advent of single-cell sequencing, new dimension reduction methods have been emerging, such as single-cell harmful binomial matrix decomposition (Sun et al., 2019), single-cell bimodal clustering analysis (Kim et al., 2018), and new feature methods to speed up the analysis and processing speed and reduce the batch effect in experiments (Li X. et al., 2020). When using single-cell sequencing to analyze data, one should treat the analysis results dialectically and conduct experimental verification in various ways. Although single-cell sequencing can provide much information, the interaction between cells, the localization of stem cells in tissues, and the epigenetic modification of genes are still unclear. Therefore, follow-up research is needed by combining single-cell sequencing with metabolomics, spatial transcriptome, and epigenetic modification of genes.

## 7. Summary

In general, single-cell sequencing provides new information and direction for the molecular basis of AIS through dimensionality reduction and bioinformatics analysis and broadens our vision of AIS cell heterogeneity and cell expression-specific genes. Identifying specific genes expressed by cells can promote the application of molecular imaging research toward monitoring dynamic disease in AIS patients. It will open up a new field for exploring the pathogenesis of AIS and drug development based on cell subtype-specific molecules and lay a foundation for further research on the human brain after AIS.

## Author contributions

XS: conceptualization and writing – original draft preparation. ML: conducting a research and investigation process and specifically performing the evidence collection. KS: presentation of the published work, specifically data presentation. YL: writing – reviewing and editing. ZG: writing – reviewing and editing and acquisition of the financial support for the project leading to this publication. All authors contributed to the article and approved the submitted version.

## Funding

This study was supported by the special fund project for doctoral training program of Lanzhou University Second Hospital (no. YJS-BD-05); the clinical Research Center for neurological diseases of Gansu Province (no. 2020-0411-SFC-0025); and Cuiying Technology Innovation Project of Lanzhou University Second Hospital (no. CY2022-QN-A05).

## Conflict of interest

The authors declare that the research was conducted in the absence of any commercial or financial relationships that could be construed as a potential conflict of interest.

## Publisher’s note

All claims expressed in this article are solely those of the authors and do not necessarily represent those of their affiliated organizations, or those of the publisher, the editors and the reviewers. Any product that may be evaluated in this article, or claim that may be made by its manufacturer, is not guaranteed or endorsed by the publisher.
